# Gloucester General Hospital

**Published:** 1831-10

**Authors:** 


### GLOUCESTER GENERAL HOSPITAL.

### *Encysted Hydrocele of the Cord, which would pass through the Abdominal Ring, and hence was mistaken for a Rupture.**

A LAD, about fifteen years of age, was brought to the hospital by a trussmaker, with a tumor of the scrotum. An apothecary had seen him, who, taking the complaint for a rupture, had ordered, and he had worn, a truss: but it appeared that his friends were dis-

* Ibid.

satisfied with the inefficiency of the instrument, and wished to have the opinion of a hospital surgeon.

An oblong tumor, between three and four inches in length, occupied the right side of the scrotum, but it did not reach to the bottom of it. It was more tense, and appeared larger, when the patient was erect; was easily diminished by pressure towards the ring; would finally pass through it, and appear reduced like a common scrotal hernia: but a close observation detected it to have lodged above the ring, in the direction of the spermatic cord, where it formed a distinct swelling. No pressure there would carry it further into the abdomen; but, upon withdrawing all pressure in that direction, the tumor reappeared in the scrotum.

The testis and spermatic cord could be distinguished at the lower part of the scrotum to be independent of the tumor, to which the act of coughing did not communicate any impulse. It was transparent when a candle was employed; there was a feeling of fluctuation, as though a fluid was contained in a very thick cyst, which appeared not to be quite full; this cyst was plainly traceable from the scrotum to the ring, its parietes feeling and slipping under the finger and thumb like an intestine. Perhaps the pressure of the truss, by inducing some absorption of the fluid within this cyst, and extending and spreading out its own parietes, gave both this character of looseness in its texture, and of its being partially filled.]

The facility with which the spermatic cord could be felt after the tumor had descended from the ring, and no impulse being given to the swelling by coughing, sufficiently distinguished it from hernia; whilst the distinct independent position of the testis, (somewhat behind the swelling,) and the obscure fluctuation, with the other characters already described, clearly marked this tumor to be an encysted tumor, or hydrocele of the cord. On the cyst being opened with a lancet, it was found to be at least a quarter of an inch thick, and containing about two ounces of bloody coloured fluid. The wound was enlarged by a bistoury to the extent of two inches, and a portion of the cyst cut away; in the act of doing which, it was found firmly adherent to the spermatic cord throughout the whole length. Considerable suppuration followed, with a degree of inflammation of the testis; but in seven weeks the patient was discharged cured, with some thickening of the spermatic cord remaining.

In recording a case of this kind, Dupuytren relates two examples of hydrocele of the tunica vaginalis, which were operated upon in a Parisian hospital, and, in consequence of the communication with the abdomen being open, the stimulating injection passed into the peritoneum. One of these patients died of peritonitis; the other escaped.

The relation of such a fact as the foregoing must lead to a belief that where any doubt exists, in a case of supposed hydrocele of the spermatic cord, as to its real nature, that carefully opening the tumor by incision, in the horizontal manner practised in strangulated hernia, would be the safest mode of cure. Its true nature would thus be revealed, and an opportunity afforded, on the escape of the fluid, to ascertain, with the finger, whether any outlet existed at any point of the cyst. Inflammation, enough to close this cyst, might then be excited by the insertion of a foreign body.

